# Severity and mortality of respiratory syncytial virus vs influenza A infection in hospitalized adults in China

**DOI:** 10.1111/irv.12754

**Published:** 2020-05-25

**Authors:** Yulin Zhang, Yeming Wang, Jiankang Zhao, Zhujia Xiong, Yanyan Fan, Wang Zhang, Xiaohui Zou, Chunlei Wang, Jiajing Han, Binbin Li, Binghuai Lu, Bin Cao, CAP-China Network

**Affiliations:** ^1^ Department of Pulmonary and Critical Care Medicine Laboratory of Clinical Microbiology and Infectious Diseases Center for Respiratory Diseases National Clinical Research Center of Respiratory Diseases China‐Japan Friendship Hospital Beijing China; ^2^ Clinical Center for Pulmonary Infections Capital Medical University Beijing China; ^3^ Tsinghua University‐Peking University Joint Center for Life Sciences Beijing China

**Keywords:** bacterial co‐infection, cardiovascular complications, in‐hospital mortality, viral infection

## Abstract

**Background:**

Respiratory syncytial virus (RSV) is an important cause of medically attended acute respiratory illnesses in older adults but awareness of the relevance of RSV in older people remains lower than that of influenza, which exhibits similar clinical characteristics to those of RSV.

**Objectives:**

This study was performed to assess the clinical significance of RSV in respiratory samples from hospitalized adults.

**Methods:**

Characteristics and outcomes in adults (≥18 years) hospitalized for RSV infection (n = 51) were compared with a cohort hospitalized for influenza A infection (n = 279) in a single‐center retrospective cohort study in Beijing, China.

**Results:**

Respiratory syncytial virus patients were slightly older, with no significant differences in underlying chronic conditions. Lower respiratory tract infection and cardiovascular complications were more frequent (*P* < .05) in RSV patients. Rates of mortality in the RSV cohorts were significantly higher within 30 days (13.7% vs 5.0%, *P* = .019) and 60 days (17.6% vs 7.5%, *P* = .021). Bacterial co‐infection in respiratory samples was associated with reduced survival among RSV patients (log rank, *P* = .013).

**Conclusions:**

Respiratory syncytial virus is a common cause of serious illness among hospitalized adults in China with greater mortality than influenza A. Increased awareness and the availability of antiviral agents might increase the scope for successful management.

## 1 INTRODUCTION

Respiratory syncytial virus (RSV) used to be known primarily as a respiratory pathogen of young children and many laudable projects such as the World Health Organization RSV surveillance platform focus on pregnant women and young children.^1^ However, in recent decades, awareness has grown of the importance of RSV infection to the health of older adults. In the United States, RSV infections occur at an annual rate of up to 10% in older adults, a rate which can exceed that observed for influenza in this population group.^2^ In the older adult population, RSV infection can have serious consequences: RSV is responsible for around 12% of all medically attended acute respiratory illnesses in older adults^3^ and the incidence of RSV‐associated hospitalization increases with age.^4^ Notably, whereas earlier data used a cutoff point of ≥65 years to define “older adult,”^2^ a recent study suggested that increased risk of severe RSV disease may commence at early as at 50 years of age.^5^ Among older adults hospitalized with RSV, a mortality rate of 6%‐8% has been reported.^3^ RSV infection was shown to lead to severe lower respiratory complications and even respiratory failure in elderly in Hong Kong, with a mortality rate up to 11.9% within 60 days.^6^ It is likely that even these alarming numbers represent an underestimation of the burden of RSV infection in older adults.^7^

Despite these numbers, awareness of the relevance of RSV in older people remains lower than that of influenza, which exhibits similar clinical characteristics to those of RSV and which has been recognized for generations as cause of severe morbidity and mortality in older adults.^8^ The need for awareness and distinction between the two diseases is illustrated by the fact that some 200 000 hospitalizations annually are associated with RSV infection compared with 300 000 hospitalizations secondary to influenza in the same population.^4^, ^5^

Low awareness is also reflected in a dearth of international data. Most of the available studies were performed in the United States. Particularly for China, with the world's largest population and an increasing proportion of elderly individuals, more data are urgently needed on the prevalence, clinical manifestations, complications, and outcomes of severe RSV infections in hospitalized adults.^6^, ^9^, ^10^ The recent progress on antiviral treatments for RSV^11^, ^12^ has given such data unprecedented relevance to clinicians.

We performed a retrospective single‐center study of a large cohort of adults hospitalized with laboratory‐confirmed RSV infections in Beijing, China, between January 2017 and June 2018. Data were gathered on characteristics, complications, and outcomes and used to compare with patients admitted for influenza A virus infection between August 2017 and June 2018.

## 2 METHODS

### 2.1 Study population

This retrospective cohort study analyzed patients aged ≥18 years admitted to the China‐Japan Friendship Hospital, Beijing with laboratory‐confirmed RSV and FA infection in 2017‐2018. A total of 51 RSV‐infected patients between January 2017 and June 2018 were enrolled in this study. All influenza A patients admitted to the center between August 2017 and June 2018 were used as the comparator group, excluding the patients who had a mixed infection of influenza A virus and RSV. The study was approved by the China‐Japan Friendship Hospital Medical Ethical Committee.

### 2.2 Clinical data collection and definitions

Electronic and written medical records were reviewed for all subjects. Data collected included demographic details, comorbid illnesses, presenting symptoms and signs, antiviral and antibiotic use, corticosteroid treatments received (intravenous or oral steroids), intensive care unit (ICU) admission, hospital length of stay, occurrence of complications, requirement for ventilatory support, exacerbation of chronic conditions, and all‐cause death within 30 days and 60 days. Medical complications associated with RSV infection were defined as a new or exacerbated medical condition as confirmed by laboratory and radiographic studies. Lower respiratory complications were defined as radiologically confirmed pneumonia or exacerbation of asthma/bronchitis/chronic obstructive pulmonary disease (COPD). Cardiovascular complications were defined as the occurrence or exacerbation of cardiac symptoms (coronary syndrome, arrhythmia, myocarditis, and decompensated heart failure) and/or acute cerebrovascular events.^13^, ^14^ Bacterial superinfection is defined as the isolation of one or more bacterial pathogen from nasopharyngeal swabs, sputum, bronchoalveolar lavage fluid and/or blood and/or urine samples.

### 2.3 Virus identification

Respiratory syncytial virus and influenza A virus infection were confirmed by analysis of nasopharyngeal swabs, sputum, bronchoalveolar lavage fluid and/or blood and/or urine samples using RSV Nucleic Acid Detection Kit (Liferiver) and Influenza A Virus Nucleic Acid Detection kit (Liferiver), respectively.^15^

### 2.4 Statistical analysis

Categorical variables are presented as frequencies and percentages. Continuous variables are described as mean, standard deviation, and range. Comparisons of proportions were performed with chi‐square and Fisher's exact tests; continuous variables were compared using Student's *t* test. All probabilities were 2‐tailed, with statistical significance defined as *P* ≤ .05. Binary logistic regression was performed to estimate the odds ratio (OR) and 95% confidence interval (CI) for clinical hospitalization outcomes in RSV‐infected patients compared with influenza A virus‐infected cohorts. Survival curves were generated using the Kaplan‐Meier method and compared using the log‐rank test. All analyses were performed using PASW Statistics software, version 18.0.

## 3 RESULTS

### 3.1 Study population

**FIGURE 1:**
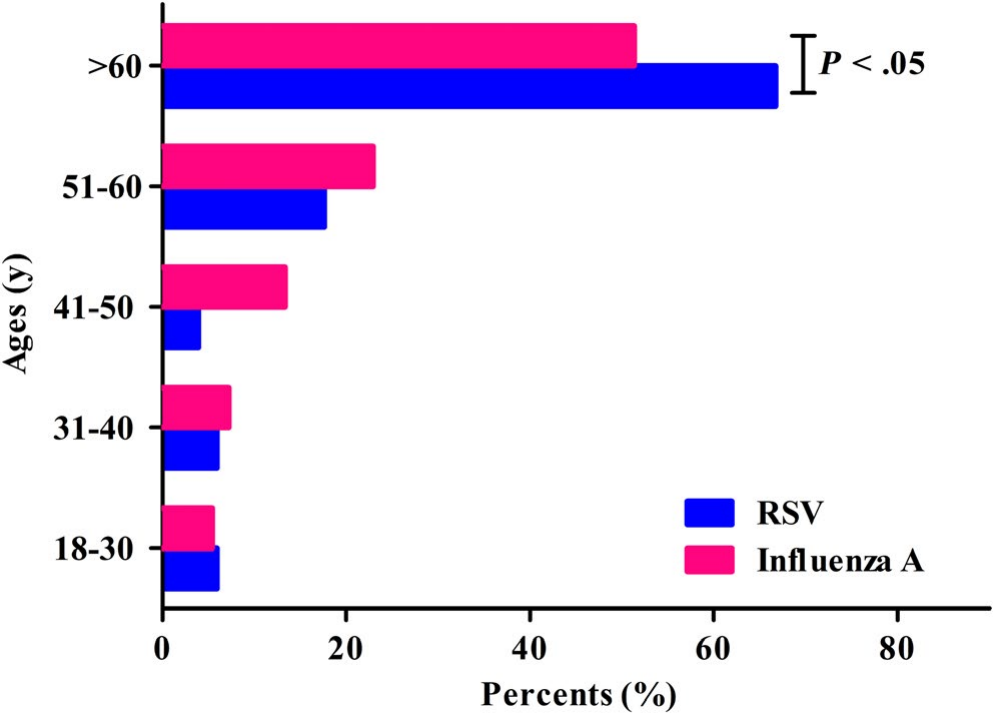
Age distribution of hospitalized adults with RSV and influenza A infection

Demographic characteristics and comorbidities prior to admission of all hospitalized patients are presented in Table 1. The proportions of women and patient smoking status were similar in the two cohorts. The median ages of RSV and influenza A virus‐infected patients were 64.1 years (SD 15.6, range 21.0‐85.0) and 60.2 years (SD 16.3, range 19.0‐94.0), respectively (*P* > .05, chi‐square test). The proportion of subjects aged >60 years was significantly greater in the RSV cohort than in the influenza A virus cohort: 66.7% vs 51.3% *P* = .042, (chi‐square test; Figure 1). There were no significant differences between the groups in rates of comorbid conditions at admission although cardiac disease, respiratory disease, and cerebrovascular disease were more common in RSV‐infected populations than those in influenza A‐infected cohorts (Table 1).

**TABLE 1:** Baseline characteristics of hospitalized adults with RSV and Influenza A infection

Patient characteristics	RSV (N = 51)	Influenza A (N = 279)	*P‐*value^a^
Age (years) at admission (mean, sd, range)	64.1, 15.6, 21.0‐85.0	60.2, 16.3, 19.0‐94.0	.116
18‐30 y (%)	3 (5.9)	15 (5.4)	
31‐40 y (%)	3 (5.9)	20 (7.2)	
41‐50 y (%)	2 (3.9)	37 (13.3)	
51‐60 y (%)	9 (17.6)	64 (22.9)	
>60 y (%)	34 (66.7)	143 (51.3)	
Male (%)	28 (54.9)	146 (52.3)	.735
Smoking			
Non‐smoker (%)	48 (94.1)	230 (82.4)	
Current smoker (%)	3 (5.9)	49 (‐17.6)	.058
Comorbidities prior to admission	47 (92.2)	231 (82.8)	.139
Cardiac disease (%)	19 (37.3)	68 (24.4)	.055
COPD (%)	7 (13.7)	22 (7.9)	.176
Bronchial asthma (%)	2 (3.9)	4 (1.4)	.233
Hypertension (%)	20 (39.2)	120 (43.0)	.614
Chronic kidney disease (%)	4 (7.8)	21 (7.5)	1.000
Any solid cancer (%)	5 (9.8)	28 (10.0)	.960
Diabetes (%)	13 (25.5)	83 (29.7)	.538
Cerebrovascular disease (%)	14 (27.5)	32 (11.5)	.002

Abbreviations: COPD: chronic obstructive pulmonary disease; RSV, respiratory syncytial virus; sd, standard deviation.

^a^ *P*‐value from chi‐square test, *t* test, Fisher's exact test, as appropriate.

### 3.2 Clinical presentation and outcomes

Clinical symptoms and outcomes in the cohorts are presented in Table 2. Fever, cough, and sputum production were the most frequent presenting signs in both cohorts, but RSV cases were less likely than influenza A cases to report fever (*P* < .001; chi‐square test) and cough (*P* = .026; chi‐square test). The rates of bacterial superinfection in each kind of samples (respiratory samples or blood samples or urine samples) were similar between RSV‐infected patients and influenza A patients (*P* > .05; chi‐square test). The median time from admission to diagnosis of RSV infection was longer than for influenza A virus infections: 4 vs 3 days, *P* = .049 (*t* test).

**TABLE 2:** In‐hospital characteristics of hospitalized adults with RSV and Influenza A infection

Patient characteristics	RSV (N = 51)	Influenza A (N = 279)	*P*‐value^a^
Symptoms			
Fever (%)	32 (62.7)	242 (86.7)	<.001
Cough (%)	34 (66.7)	225 (80.6)	.026
Sputum production (%)	32 (62.7)	192 (68.8)	.393
Hemoptysis (%)	2 (3.9)	15 (5.4)	.930
Myalgia (%)	5 (9.8)	53 (19.0)	.113
Weakness (%)	8 (15.7)	71 (25.4)	.133
Days from admission to diagnosis (median, IQR)	4, 1‐9	3, 1‐6	.049
Any antiviral drug use during hospitalization (%)	21 (41.2)	242 (86.7)	<.001
Oseltamivir use (%)	12 (23.5)	242 (86.7)	<.001
Ribavirin use (%)	8 (15.7)	0 (0.0)	*<*.001
Any antibiotic drug use during hospitalization (%)	45 (88.2)	235 (84.2)	.463
Any intravenous or oral steroid use during hospitalization (%)	27 (52.9)	107 (38.4)	.051
Intravenous steroid use (%)	21 (41.2)	71 (25.4)	.021
Oral steroid use (%)	6 (11.8)	36 (12.9)	.823
Bacterial superinfection^b^ (%)			
Blood samples	3 (5.9)	6 (2.2)	.300
Respiratory samples	16 (31.4)	98 (35.1)	.604
Urine samples	4 (7.8)	8 (2.9)	.181
Complication/outcome			
Lower respiratory tract complications^c^ (%)	32 (62.7)	126 (45.2)	.021
Cardiovascular complications^d^ (%)	26 (51.0)	96 (34.4)	.024
Pneumonia (%)	30 (58.8)	107 (38.4)	.006
Need for intensive care (%)	12 (23.5)	70 (25.1)	.813
Need for invasive mechanical ventilation (%)	12 (23.5)	48 (17.2)	.282
In‐hospital mortality (%)	9 (17.6)	21 (7.5)	.021
30‐d mortality (%)	7 (13.7)	14 (5.0)	.019
60‐d mortality (%)	9 (17.6)	21 (7.5)	.021
Time to death (days) (median, IQR)	10, 8.5‐14	11, 8‐18.5	.762
Duration of hospitalization for survivors (days) (median, IQR)	15, 13‐22	14, 10‐19	.148

Abbreviations: IQR, interquartile range; RSV, respiratory syncytial virus.

^a^ *P*‐value from chi‐square test, *t* test, Fisher's exact test, as appropriate.

^b^ Bacterial superinfection is defined as the isolation of one or more bacterial pathogen from respiratory samples (nasopharyngeal swabs, sputum, and bronchoalveolar lavage fluid) and/or blood and/or urine samples.

^c^ Lower respiratory complications included radiologically confirmed pneumonia or exacerbation of asthma/bronchitis/chronic obstructive pulmonary disease [13,14].

^d^ Cardiovascular complications included the occurrence or exacerbation of cardiac symptoms (coronary syndrome, arrhythmia, myocarditis, and decompensated heart failure) and/or acute cerebrovascular events [13,14].

During hospitalization lower respiratory tract complications occurred in 62.7% of RSV cases and 45.2% of influenza A cases, respectively (*P* = .021; chi‐square test). Cardiovascular complications during hospitalization were also more frequent in the RSV group than in the cohort with influenza A virus infection: 51.0% vs 34.4%, *P* = .024 (chi‐square test), as was pneumonia: (58.8% vs 38.4%, *P* = .006; chi‐square test).

Use of antibiotics as well as of intravenous or oral corticosteroids during the hospitalization period was similar in the two cohorts. Among RSV cases, oseltamivir was prescribed significantly less commonly to RSV‐infected than to influenza A‐infected patients (23.5% vs 86.7%, *P* < .001; chi‐square test) but whole ribavirin was prescribed only for RSV infections (15.7% vs 0.0%, *P* < .001; chi‐square test). Use of invasive mechanical ventilation was similar in both cohorts. There were no differences in rates of ICU admission between the cohorts.

Rates of mortality in the RSV cohorts were significantly greater than that for influenza A‐infected patients within 30 days (13.7% vs 5.0%, *P* = .019; chi‐square test) and 60 days (17.6% vs 7.5%, *P* = .021; chi‐square test) respectively. There were no differences in median time from admission to death between the groups nor in the median duration of hospitalization for survivors. In the binary logistic regression analyses, the odds of hospitalization outcomes (cardiovascular complications, pneumonia, lower respiratory tract complications, the need for invasive mechanical ventilation and 60‐day mortality) in RSV cases were higher than in those hospitalized with influenza A infection, but the 95% CI crossed the boundary for all variables except for cardiovascular complications (Table 3).

**TABLE 3:** Binary logistic regression analyses associated with clinical hospitalization outcomes in hospitalized adults with RSV and Influenza A infection

Hospitalization outcomes^a^	OR	95% CI
Lower respiratory tract complications	1.4	0.6‐3.3
Pneumonia	1.9	0.8‐4.6
Cardiovascular complications	2.7	1.2‐6.2
Need for invasive mechanical ventilation	1.4	0.6‐3.4
60‐d mortality	1.7	0.6‐4.6

Abbreviations: CI, confidence interval; OR, odd ratio.

^a^ Hospitalization outcomes included lower respiratory tract complications, pneumonia, cardiovascular complications, the need for invasive mechanical ventilation and 60‐d mortality.

### 3.3 Analysis of RSV cases with fatal outcomes

**FIGURE 2:**
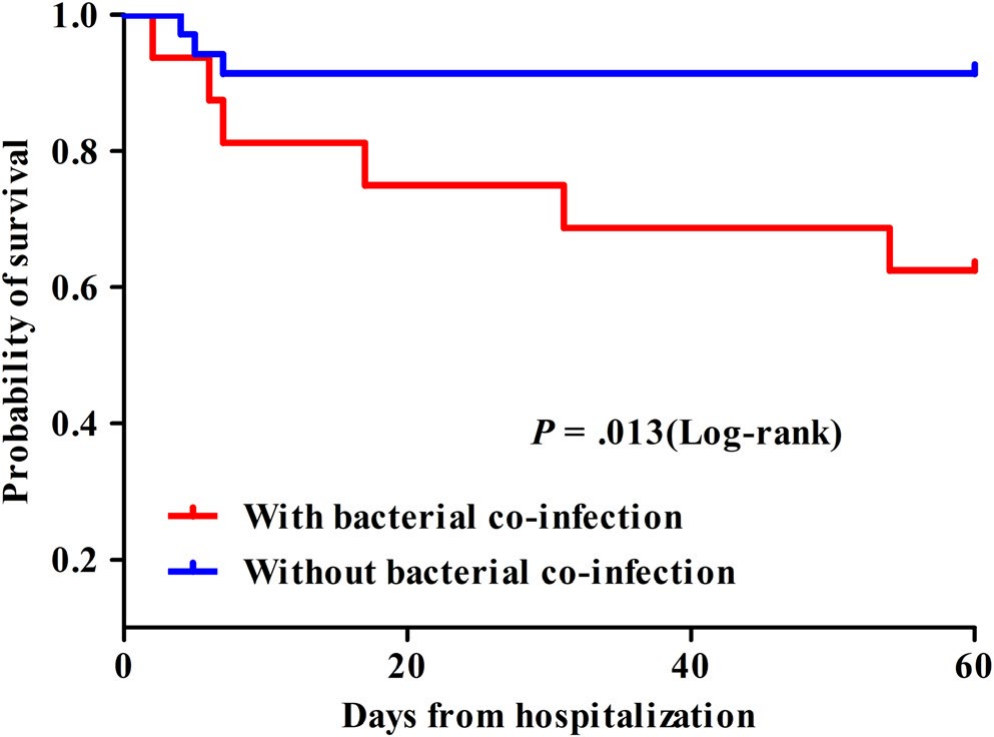
Kaplan‐Meier survival curves for patients with RSV infection with (n = 16) and without (n = 35) bacterial co‐infection in respiratory samples (nasopharyngeal swabs, sputum, or bronchoalveolar lavage fluid), respectively

Nine patients with RSV infection died during hospitalization. A comparison with survivors showed no differences in sex, comorbidities, blood biochemical indices, symptoms, and signs; notably though, bacterial superinfection in respiratory samples (nasopharyngeal swabs, sputum, or bronchoalveolar lavage fluid) was more common among non‐survivors than that in survivors (*P* = .021, chi‐square test; Table 4) and was showed to be related to lower survival (Figure 2). Mortality within 60 days in patients with bacteria and RSV co‐infection in respiratory samples was up to 37.5%. Injected or oral corticosteroid use was more frequent in deceased than in surviving patients (88.9% vs 45.2%, *P* = .044 (chi‐square test) as was use of invasive mechanical ventilation (66.7% vs 14.3%, *P* = .003; chi‐square test) and ICU admission (66.7% vs 14.3%, *P* = .003; chi‐square test; Table 4). Survivors tended to have less cardiac disease and lower respiratory tract complications, but these differences did not reach statistical significance.

**TABLE 4:** Patients characteristics of RSV‐infected adults with and without survivor

Patient characteristics	Dead cases (N = 9)	Survivors (N = 42)	*P*‐value^a^
Male (%)	5 (55.6)	23 (54.8)	1.000
Age (years) (mean, sd, range)	73.3, 9.3, 54.0‐84.0	62.1, 16.1, 21.0‐85.0	.049
Smoking			
Current smoker (%)	0 (0.0)	3 (7.1)	1.000
Current non‐smoker (%)	9 (100.0)	39 (92.9)	
Comorbidities prior to admission			
Hypertension (%)	4 (44.4)	16 (38.1)	1.000
Diabetes (%)	2 (22.2)	11 (26.2)	1.000
Cerebrovascular disease (%)	4 (44.4)	10 (23.8)	.397
Chronic kidney disease (%)	2 (22.2)	2 (4.8)	.278
Any solid cancer (%)	1 (11.1)	4 (9.5)	1.000
COPD (%)	2 (22.2)	5 (11.9)	.778
Bronchial asthma (%)	0 (0.0)	2 (4.8)	1.000
Complication/outcome			
Cardiac disease^b^ (%)	5 (55.6)	14 (33.3)	.384
Pneumonia (%)	7 (77.8)	23 (54.8)	.368
Lower respiratory tract complications^c^ (%)	7 (77.8)	25 (59.5)	.517
Symptoms and signs			
Temperature (mean, sd, range)	38.6, 1.4, 36.4‐40.0	37.6, 1.2, 36.0‐41.0	.025
Cough (%)	7 (77.8)	27 (64.3)	.697
Hemoptysis (%)	0 (0.0)	2 (4.8)	1.000
Sputum production (%)	6 (66.7)	26 (61.9)	1.000
Myalgia (%)	1 (11.1)	4 (9.5)	1.000
Weakness (%)	3 (33.3)	5 (11.9)	.272
Need for invasive mechanical ventilation (%)	6 (66.7)	6 (14.3)	.003
Bacterial superinfection^d^ (%)			
Blood samples	0 (0.0)	1 (2.4)	1.000
Respiratory samples	6 (66.7)	9 (21.4)	.021
Urine samples	2 (22.2)	2 (4.8)	.278
Need for intensive care (%)	6 (66.7)	6 (14.3)	.003
Any antiviral drug use during hospitalization (%)	4 (44.4)	17 (40.5)	1.000
Oseltamivir use (%)	4 (44.4)	8 (19.0)	0.231
Ribavirin use (%)	0 (0.0)	8 (19.0)	.322
Any injection or oral steroid use during hospitalization (%)	8（88.9）	19（45.2）	.044
Any antibiotic drug use during hospitalization (%)	9（100.0）	36（85.7）	.575
Blood biochemical indexes			
Serum alanine aminotransferase Concentration, IU/L (mean)	606.5	49.9	.370
Bilirubin (mean)	22.2	14.9	.557
Serum creatinine	203.3	102.5	.425
Glucose	7.2	7.3	.933

Abbreviations: COPD: chronic obstructive pulmonary disease; IQR, interquartile range; RSV, respiratory syncytial virus; sd, standard deviation.

^a^ *P*‐value from chi‐square test, *t* test, Fisher's exact test, as appropriate;

^b^ Cardiac disease included the occurrence or exacerbation of cardiac symptoms (coronary syndrome, arrhythmia, myocarditis, and decompensated heart failure) [13,14];

^c^ Lower respiratory complications included radiologically confirmed pneumonia or exacerbation of asthma/bronchitis/chronic obstructive pulmonary disease [13,14];

^d^ Bacterial superinfection is defined as the isolation of one or more bacterial pathogen from respiratory samples (nasopharyngeal swabs, sputum, and bronchoalveolar lavage fluid) and/or blood and/or urine samples.

## 4 DISCUSSION

With RSV‐specific antiviral therapy advancing in clinical development, the question of differentiating RSV infection from that of influenza in adult populations will likely become highly relevant to care decisions worldwide.^12^, ^16^ The current retrospective study is the first, to our knowledge, to describe and compare populations of adults hospitalized with RSV or influenza A virus infection in China. The results show RSV infection to be an important cause of morbidity and mortality in this, the largest population in the world. The comparisons with influenza A are relevant to clinicians faced with adults hospitalized with respiratory tract infection beyond China.

Some observations such as the greater age, higher rates of complications, and greater mortality of RSV‐infected subjects compared with influenza A virus infection confirm the serious nature of RSV that has been reported from other countries, mostly the United States.^17^, ^18^, ^19^, ^20^, ^21^ Additionally, in consistent with the previous studies, higher proportions of patients with cardiac disease, respiratory disease, and cerebrovascular disease were observed in RSV‐infected populations than those in influenza A‐infected cohorts although there is no significant difference.^20^, ^21^ The higher underlying conditions might be the cause of higher rates of lower respiratory tract infection and cardiovascular complications in these cohorts. Other findings diverge from earlier reports. The RSV‐infected hospitalized adults in the present study were younger than those described in other studies,^6^, ^19^, ^20^ but the mortality was higher. These findings may reflect low awareness of the seriousness of RSV infection, as has been observed in other countries.^22^, ^23^ Similarly, although RSV patients were twice as likely as those with influenza A infection to present with lower respiratory tract and cardiovascular complications, there were no differences in the use of inhaled, oral or intravenous steroids, ICU admission, and invasive mechanical ventilation in the hospital.

We found a high rate of bacterial co‐infection in respiratory samples among non‐survivors with RSV infection and a likely correlation with mortality. As the data are from a retrospective analysis, a causal connection between bacterial infection and excess mortality cannot be definitively demonstrated; this would need further studies. It is possible that the respiratory tract microbiome influences host responses to RSV, modulating inflammation, and disease severity^24^ although the immunopathogenesis of co‐infection remains unknown. Whatever the causal relationship, the findings support the recommendation that RSV‐infected patients with any bacterial infection during hospitalization should be promptly identified and treated.^25^

The differences in presentation at admission between RSV and influenza A infection are of interest. Fever and cough were less common among RSV cases, but rates of lower respiratory tract and cardiovascular complications, especially pneumonia, were greater in the RSV‐infected population. The latter complications may partially explain the higher rates of mortality in this group. It is also possible that lower respiratory tract disease progression is more rapid in RSV infection, although further research into the mechanisms and natural history may be necessary.^19^, ^26^

The seasonal pattern of RSV infection in children in China has recently been shown to be very similar to those in the United States,^27^ and it is reasonable to assume that this would also be the case for adult disease. The development of efficacious interventions against RSV should be a high priority as they could reduce mortality and morbidity, and burdens on the healthcare system. Furthermore, if early identification and diagnosis of RSV infection in hospitalized adults with bacterial co‐infection enabled the timely implementation of appropriate therapies to reduce complications, this would reduce mortality, morbidity, and healthcare costs. An economic analysis in the United States estimated the average cost of RSV hospitalizations to be more than twice that of influenza A.^28^ No economic data are available for our cohort but a substantial economic burden can be inferred from the demonstrated severity of the RSV‐infected population.

There are limitations to this study. It was a single‐center analysis with modest sample size, and the features of the setting may not be representative of China as a whole. As a retrospective study, causation, for example, between bacterial co‐infection and mortality cannot be definitively determined. There was no analysis of RSV genotypes, which would be important for future epidemiological studies as well as to possibly assess future anti‐RSV therapies.^29^

In conclusion, RSV infection is a common cause of serious illness among hospitalized Chinese adults, with greater morbidity and mortality than influenza A virus infection. Greater awareness of the serious nature of RSV infection among healthcare professionals would enable adult RSV‐infected patients, particularly those with bacterial infection or prior cardiac and pulmonary disease to be recognized in time and given appropriate treatments on admission.^30^ If recent reports of successful antiviral treatment for RSV^12^ are confirmed in further clinical trials these needs will take on a heightened relevance.

## CONFLICT OF INTEREST

The authors declare that they have no conflict of interest.

## AUTHOR CONTRIBUTIONS

**Yulin Zhang:** Data curation (Equal); Formal analysis (Lead); Funding acquisition (Supporting); Investigation (Equal); Methodology (Equal); Project administration (Equal); Validation (Equal); Visualization (Equal); Writing (original) draft (Lead). **Yeming Wang:** Data curation (Equal). **Jiankang Zhao:** Investigation (Equal); Resources (Equal); Software (Equal). **Zhujia Xiong:** Methodology (Equal), Resources (Equal); Software (Equal); Validation (Equal). **Yanyan Fan:** Resources (Equal); Visualization(Equal). **Wang Zhang:** Resources (Equal). **Xiaohui Zou:** Resources (Equal). **Chunlei Wang:** Data curation (Equal); Resources (Equal). **Jiajing Han:** Resources (Equal). **Binbin Li:** Resources (Equal). **Binghuai Lu:** Project administration (Equal); Supervision (Lead); Validation (Equal). **Bin Cao:** Conceptualization (Lead); Funding acquisition (Lead).
